# Sculptors of cerebellar fissures and their potential as therapeutic targets for cerebellar dysfunction

**DOI:** 10.3389/fncel.2025.1608185

**Published:** 2025-06-05

**Authors:** Chiu-Lun Shen, Yu-Young Tsai, Woan-Yuh Tarn

**Affiliations:** ^1^Institute of Biomedical Sciences, Academia Sinica, Taipei, Taiwan; ^2^Department of Biological Sciences, Columbia University in the City of New York, New York, NY, United States

**Keywords:** cerebellar foliation, cerebellar fissure, cerebellar disorder, developmental signaling, BDNF

## Abstract

The cerebellum plays an important role in both motor control and cognition. The cerebellar cortex is neuron-rich and composed of characteristic folia and fissures. Defective cerebellar development leads to movement disorders and developmental delay. During early morphogenesis, cellular signaling programs orchestrate simultaneous cerebellar growth and foliation. Aberrant signaling causes various degrees of cerebellar hypoplasia. Based on mouse genetic studies, we discuss several developmental signaling pathways that drive cerebellar morphogenesis. Notably, hypoplasia of vermal lobules VI-VII has been linked to autism spectrum disorder and is in part attributed to brain-derived neurotrophic factor (BDNF)/tropomyosin receptor kinase B signaling. This review also discusses how BDNF biogenesis is critical for cerebellar foliation and whether restoring BDNF signaling could reverse cerebellar developmental disorders.

## 1 Introduction

The cerebellum is a neuron-rich structure residing at the base of the brain, connecting with the cerebrum, the brainstem and the spinal cord. Long recognized for its central role in movement control and coordination, the cerebellum is increasingly implicated in cognitive functions, including social behavior, reward circuitry, emotional and language processing (Carta et al., 2019; Wagner and Luo, 2020; Stoodley et al., 2021). A range of neurodevelopmental disorders, such as Joubert syndrome and autism spectrum disorders (ASD), arises from disruptions in cerebellar development of varying severity. Unraveling how genetic disruptions affect cerebellar morphogenesis is crucial. To gain insights into developmental origins of brain disorders, we conducted an extensive literature and database search to identify gene deficiencies that affect cerebellar fissure formation in mouse models. Through such an analysis, this review offers unique insights into how cerebellar fissure morphogenesis is controlled by cellular signaling programs. Moreover, we discuss the potential of small molecule compounds that modulate signaling pathways for reversing cerebellar developmental disorders, particularly hypoplasia of the vermal lobules VI and VII associated with ASD.

## 2 The cerebellum and its functional architecture

**Figure 1:**
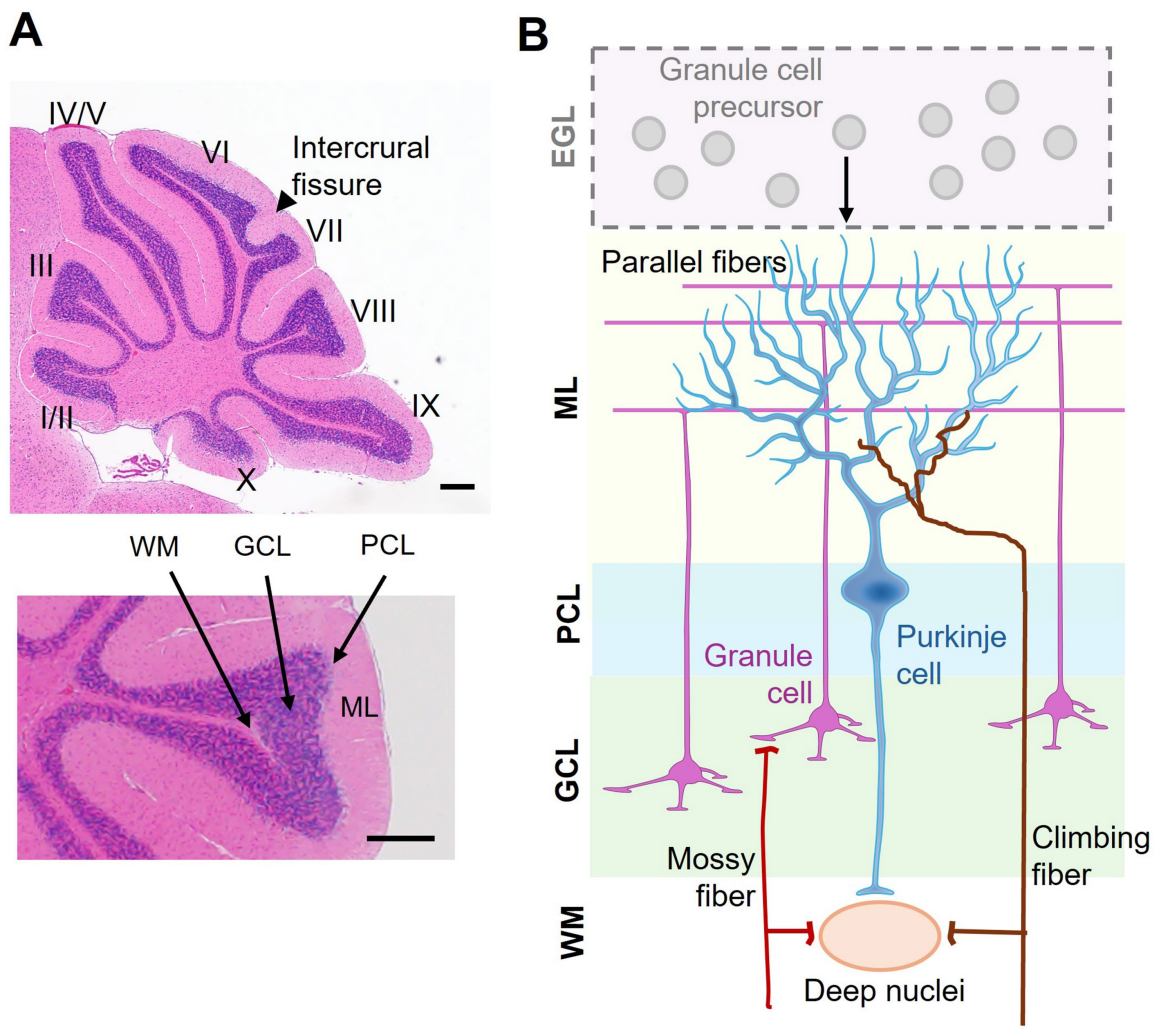
Laminar organization of the cerebellum. **(A)** A sagittal view of a mouse cerebellum stained with hematoxylin and eosin. Upper: the 10 lobules (I–X) and the fissure between lobules VI and VII, namely the intercrural fissure, are indicated. Lower: distinct layers of the cerebellar cortex, including the molecular layer (ML), Purkinje cell layer (PCL), granule cell layer (GCL), and white matter (WM). **(B)** Schematic representation of cerebellar cortical organization and connectivity. During early development, granule cell precursors (GCPs) proliferate in the external granule layer (EGL). The EGL disappears after granule cells (GCs) radially migrate inward to form the GCL in the mature cerebellum. The ML contains Purkinje cell dendrites and granule cell axons (i.e., parallel fibers). The PCL consists of a monolayer of Purkinje cells (PCs), acting as the output neurons of the cortex. Excitatory inputs to the cerebellum include mossy fibers, which relay sensory and motor information via granule cells, and climbing fibers, which form synaptic connections with PCs. The deep nuclei located in the WM integrate inhibitory signals from PCs and excitatory afferents, serving as the principal output hub of the cerebellum. Scale bar: 200 μm.

The cerebellum consists of two hemispheres connected by a narrow midline region called the vermis. The foliation pattern is symmetrical to the midline and gives rise to 10 lobules I–X that run perpendicular to the anterior–posterior axis along the vermis (Sillitoe and Joyner, 2007; Farini et al., 2021) (Figure 1A, upper panel). The cerebellar cortex has a laminar organization, i.e., the molecular layer (ML), Purkinje cell layer (PCL), and granule cell layer (GCL), going from the outer to inner direction (Figure 1A, lower panel). Nearly 99% of cerebellar neurons are granule cells (GCs). During development, GC precursors (GCPs) proliferate at the external granule layer (EGL), which is an outermost and transient layer (Figure 1B). After mitosis, GCs migrate inward to populate the GCL (for the detail, see below). The somata of Purkinje cells (PCs) form a monolayer called PCL. The molecular layer (ML) is largely cell-free but contains neuronal microcircuits, including GC axons, PC dendrites, and their synapses, as well as interneurons and glia (Apps and Hawkes, 2009) (Figure 1B). In the ML, axons of GCs ascend from the soma, forming parallel fibers which have extensive connections with dendrites of PCs. Besides, two major excitatory afferents, *mossy fibers* and climbing fibers, from other brain areas and spinal cord terminate in GCs (Legué et al., 2016). Embedded within the white matter under the cerebellar cortex, the deep cerebellar nuclei primarily receive afferents from the GABAergic PCs and generate the principal outputs of the cerebellum (Figures 1A,B). Precise organization of neurites and their connectivities constitute the cerebellar circuitry (Legué et al., 2016).

The cerebellum processes information from functionally diverse regions of the cerebral cortex to its support motor, cognitive, and affective functions (Stoodley et al., 2012). An early study reported that birth date-related PC clustering may correlate with functional compartmentalization along the mediolateral axis in the adult cerebellum (Hashimoto and Mikoshiba, 2003). Task-based neuroimaging studies have more recently revealed distinct functional territories of the cerebellum, i.e., sensorimotor (lobules II–VI, VIIIB), vestibular (lobules IX–X), oculomotor (lobules VI–VII, IX–X), visual (lobule VI), and auditory (lobules V–VI) zones (Stoodley and Schmahmann, 2018; Raymond and Medina, 2018) (Figure 1A). Based on spatiotemporal transcriptomics, it is now evident that the cerebellum exhibits regional specialization physiological and anatomical properties across subregions (i.e., folia) and neuronal subtypes (Cerminara et al., 2015; Kozareva et al., 2021; Lanore et al., 2021; Sepp et al., 2024). As a prime example of non-uniform microcircuitry, the longitudinal zebrin II+/− stripes in PCs reveal molecular heterogeneity that restricts specific splicing variant expression that influences PC plasticity, firing properties, and input–output connections (Lin et al., 2020). Therefore, cerebellar development is a highly regulated and genetically influenced process, rather than a uniform one. Nevertheless, more research is needed to determine how molecular factors align with functional specialization.

## 3 Lobules VI–VII: what is special?

Gene deficiencies affect cerebellar morphogenesis to varying degrees. Certain developmental defects contribute to hypoplasia of the vermian lobules VI–VII, a feature associated with ASD (Courchesne et al., 1988). Developmentally, lobules VI and VII expand significantly in the lateral hemisphere forming crus I in the rodent cerebellum, the homologous region of human crus I/II implicated in cognitive and visuomotor functions (Sugihara, 2018). Notably, lobules VI-VII exhibited a delayed developmental timeline in comparison with that of other lobules (Legué et al., 2016). Functional topography has revealed that lobules VI and VII are embryologically and phylogenetically distinct from the anterior lobules I–V (Courchesne et al., 1988). Tract-tracing studies revealed reciprocal connection from this posterior vermal region to associative and paralimbic cortices, providing anatomical substrate for cognitive functions (Kelly and Strick, 2003). Of note, lobule VII accounts for 47.70% gray matters of the human cerebellar volume (Diedrichsen et al., 2009). *In vivo* electrophysiological studies in the mouse cerebellum underscored an interplay of intrinsic cell properties and input–output profiles underpinning a zonal distribution of various cerebellar functions. For example, mossy fiber burst inputs to GCs and PC firing rates are characteristically different for lobules VI–VII versus X. This difference in input–output regularity may support the neural processing required by distinct tasks each subregion is involved in: an oscillation-based communication with cerebral cortex (lobules VI–VII) versus an always-on mechanism for vestibular functions (lobule X) (Witter and De Zeeuw, 2015).

## 4 Cerebellar development: cortical lamination and foliation

**Figure 2:**
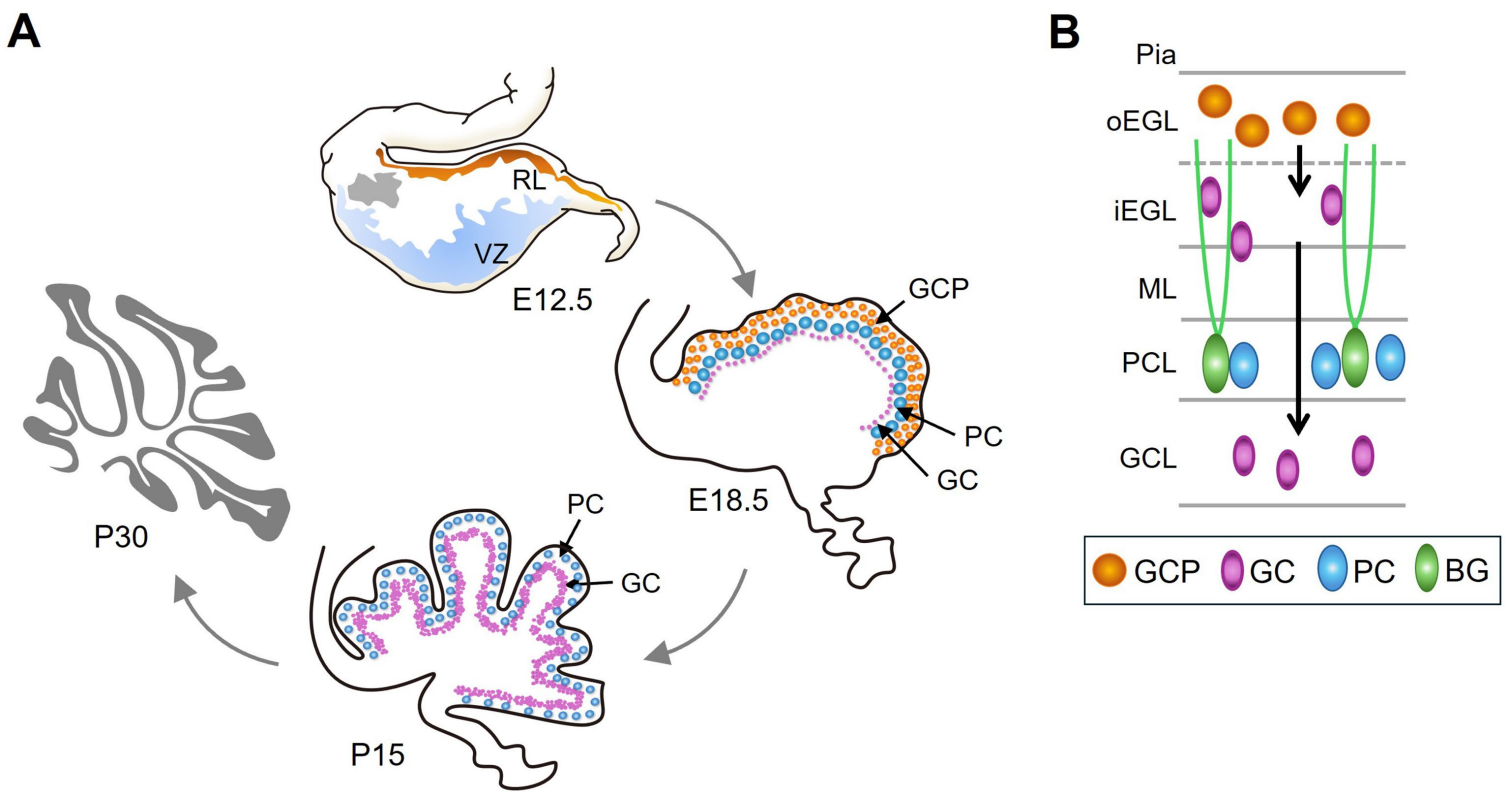
The development of the cerebellum. **(A)** Cerebellar development begins at E9 in mice. At E12.5, the cerebellar primordium forms in the roof of the fourth ventricle, consisting of two primary germinal zones: the ventricular zone (VZ) (blue) and the rhombic lip (RL) (orange). Grey indicates the nuclear transitory zone, where deep nuclear neurons develop. At E18.5, GCPs proliferate in the outermost layer, i.e., EGL (orange). PCs and postmitotic GCs (purple) are found beneath the EGL. By P15, GC maturation and migration are complete, leading to accumulation in the GCL, which is inside of the PCL. **(B)** Schematic of different cell types and its location in the developing cerebellum. GCPs proliferate at outer EGL (oEGL); after exiting the cell cycle, GCs migrate inward to inner GCL (iEGL). Subsequently, GCs migrate radially on Bergmann glial (BG) fibers to populate the interior of the cerebellum and ultimately form the GCL.

The pattern of vermis foliation is generally conserved across mammalian species. The human cerebellum begins to develop at gestational week 4 and ends around 2–3 years after birth (Carletti and Rossi, 2008; Iskusnykh and Chizhikov, 2022). In mice, cerebellar development begins at embryonic day (E) 9 and ends in the third postnatal week. The cerebellar primordium emerges in the roof of the fourth ventricle, comprising two primary germinal zones, i.e., the ventricular zone (VZ) and the rhombic lip (RL) (Leto et al., 2016) (Figure 2A, E12.5). VZ progenitor cells produce GABAergic PCs and Bergmann glia precursors. The RL produces glutamatergic GC precursors (GCPs). Foliation occurs concurrently with the formation of the cortical cell layers. Fissure formation is initiated around E16.5 when GCP expands at the outermost EGL, a transient secondary germinal zone, and move inwards to the inner EGL (Figure 2A, E18.5 and Figure 2B). GCP proliferation peaks between postnatal day 5 and 8 (P5-8). Subsequently, postmitotic GCs undergoes radial migration from the EGL across the PCL to populate the internal granule layer (IGL) (Figure 2B). The radial glia in the VZ transforms into Bergmann glia during E14.5-E18.5. Bergmann glial cells are essential for the folding of the cerebellar surfaces (Sudarov and Joyner, 2007; Leung and Li, 2018). As the anchoring points, Bergmann glia located next to the PC layer emit their radial fibers toward the pial surface for GC migration (Rahimi-Balaei et al., 2018) (Figure 2B). GC maturation and migration complete by P15, and proliferative EGL disappears (Figure 2A, P15) (Carletti and Rossi, 2008; Iskusnykh and Chizhikov, 2022). Notably, the kinetics of GC accumulation varies across different zones of the cerebellum. Maximal GC production is delayed in the lobules VI and VII compared to other zones (Legué et al., 2016). Whether such a delay results in the formation of the fissure (namely the intercrural fissure, see below) between lobules VI and VII particularly sensitive to certain cellular signals or neurotransmitters remains as an intriguing question.

Cerebellar development is orchestrated through a program of transcriptional regulation and signaling pathways. Recently, single-cell transcriptomic profiling has identified the expression of nearly 200 cell-type specific transcription factors in the developing cerebellum and determined their respective regulon activities (Sepp et al., 2024). On the basis of mutant-induced phenotypic changes (see Section 6), this review discusses signaling pathways essential for cerebellar development. As early as E8.5, Wnt1 drives the expression of FGF8, a morphogen that specifies hindbrain and controls the onset of cerebellar development (Spassky et al., 2008). Bone morphogenetic proteins (BMPs) function before the formation of the cerebellar primordium to control stem cell specification in the anterior rhombic lip (Tong et al., 2015). Notch signaling preserves a pool of neural progenitor cells in an undifferentiated state. However, Notch levels differ between daughter cells after cell division. Although high levels of Notch maintain the progenitor fate, the intermediate and low levels of Notch activities may, respectively, generate inhibitory and excitatory neurons (Zhang et al., 2021). From E17.5 to postnatal days, PCs express Sonic hedgehog (Shh) to promote GCP proliferation in the EGL (Wallace, 1999). Reciprocally, Reelin released by GCPs disperses PC clusters into the monolayer (Miyata et al., 1997). Dysregulation of these cellular signaling pathways contributes to a range of cerebellar disorders (see below).

## 5 Cerebellar malformations in human disorders

Cerebellar malformations underpin a spectrum of debilitating disorders, from ataxia to autism. Accumulating animal-based evidence highlights loss of function genes and defective signaling that give rise to abnormal cellular patterning and foliation defects underlying disease progression. In this review, we describe several cerebellar developmental disorders and their associated susceptibility genes that have been confirmed in animal studies.

### 5.1 Dandy Walker malformation

Dandy Walker malformation (DWM) is the most common cerebellar malformation in human live births, characterized by dilation of fourth ventricle and vermis hypoplasia. DWM patients exhibit symptoms ranging from intellectual disability to autism (Carletti and Rossi, 2008). Genetic aberrations of DWM have been described in a variety of genes including *ZIC1*, *ZIC4* and *FOXC1* (Grinberg et al., 2004; Aldinger et al., 2009). The phenotypes of mice with a heterozygous deletion of *Zic1* and *Zic4* closely resemble DWM (Grinberg et al., 2004). Hypomorphic *Foxc1* mutant mice exhibited similar defects in cerebellar foliation to DWM individuals with *FOXC1* locus deletions (Haldipur et al., 2017).

### 5.2 Joubert syndrome-related disorders

Characterized by ataxia and delayed development, patients with Joubert syndrome similarly suffer from cerebellar vermis hypoplasia (Valente et al., 2006). Joubert syndrome-related disorders exhibit clinical heterogeneity due to their various genetic causes, including three genes (*CEP290*, *AHI1*/*JBTS3*, *NPHP1*/*JBTS4*) and two loci (*JBTS1* and *JBTS2*) (Valente et al., 2005; Valente et al., 2006). Nevertheless, as with Joubert syndrome individuals, *Ahi1* knockout mice exhibited hypoplasia in lobules VI-VII. Ahi1 has been linked to Wnt-*β*-catenin signaling (Lancaster et al., 2009), and a partial reversal of the cerebellar defects was achieved by treating these mice with lithium, a Wnt agonist (Lancaster et al., 2011).

### 5.3 CHARGE syndrome

CHARGE syndrome is characterized by multiple organ defects and commonly associated with *CHD7* mutations (Zentner et al., 2010). Notably, individuals with CHARGE syndrome display cerebellar vermis hypoplasia and foliation defects. Similarly to CHARGE individuals, mice with *Chd7* haploinsufficiency displayed foliation defects, developmental delay, and motor deficits caused by impaired epigenomic regulation of GCP differentiation and foliation anomalies (Zentner et al., 2010; Reddy et al., 2021).

### 5.4 Autism spectrum disorder

Autism spectrum disorder (ASD) represents a heterogeneous group of disorders characterized by social deficits and repetitive behaviors. Strong correlative evidence between abnormal cerebellar development and ASD has been established. *CHD8* is one of the most frequently mutated genes in individuals with ASD (Talkowski et al., 2012; Neale et al., 2012; O'Roak et al., 2012). Ablation of *Chd8* in mouse GCP resulted in pronounced foliation defects, vermis hypoplasia, and motor defects (Kawamura et al., 2021). Other transgenic mouse studies also converge on the association between cerebellar malformation and ASD-like phenotypes (see below).

### 5.5 Spinocerebellar ataxia

Spinocerebellar ataxia (SCA) is caused by variants in many different genes. A subset of hereditary cerebellar ataxia exhibits impaired PC dendritic arborization, zebrin-II stripe degradation, and climbing fiber dysfunction (Bartelt et al., 2024). The review will not discuss SCA, since it is an adult-onset neuro-degenerative disorder.

## 6 Mouse genes critical for cerebellar foliation

Over the past three decades, studies of mouse disease models have provided substantial information regarding cerebellar development and implication for human disorders associated with cerebellar malformations (Manto and Jissendi, 2012; Butts et al., 2014; Haldipur and Millen, 2019). Moreover, our understanding of cerebellar morphogenesis may be sped up by unexpected discoveries of genes associated with disease phenotypes. A comprehensive database has compiled a total of 543 mouse genes critical for cerebellar development and 630 human mutant loci associated with cerebellar phenotypes (Ramirez et al., 2022). Recent studies using single-cell RNA-seq of the cerebellum across developmental stages and species have revealed its cellular architecture, evolutionary differences, and insights into cerebellar diseases (Cerminara et al., 2015; Carter et al., 2018; Haldipur et al., 2019; Aldinger et al., 2021). To date, we still lack a complete understanding of the mechanisms governing cerebellar morphogenesis and how their dysregulation contributes to cerebellar disorders.

To improve our understanding of cerebellar foliation, we collected mouse genes that have been reported in cerebellar foliation from Mouse Genome Informatics (MGI)^1^ and PubMed.^2^ In the MGI database, 69 genes are categorized as having abnormal cerebellar fissure morphology or lobule formation, or with reduced or absent foliation in the cerebellum (Table 1, MGI). A PubMed search for “cerebellar hypoplasia” identified 52 genes whose knockout reduces cerebellum size and 19 genes whose knockout impairs intercrural fissure formation (Table 1, PubMed). Intriguingly, only 15 genes from this search were identified in the above categories of MGI (Table 1, footnotes). PubMed also revealed that approximately 30 genes whose knockout does not significantly affect cerebellar morphology, but several of them affect PC arborization or function, such as *Bcl7a*, *Pten* and *Tsc2* (Tsai et al., 2012; Cupolillo et al., 2016; Wischhof et al., 2017).

**Table 1:** Search of genes involved in cerebellar morphogenesis.

MGI				Pubmed					
				Whole CB reduction			Icf reduction	No morphological change	
*Abl1* *Abl2* *Ahi1* *Arcn1* *B4galt2* *Bax* *Braf* *Cadps2* *Cbl1* *Ccnd1* *Ccnd2* *Cep120* *Cep290* *Cers1* *Chd7* *Chmp1a* *Ctnna2* *Dab1* *Dnm1* *Edaradd*	*Ei24* *En1* *En2* *Ercc6* *Fbxw7* *Fign* *Foxp2* *Fubp1* *Gabrb3* *Gja1* *Gli2* *Gli3* *Grid2* *Itgb1* *Kcnj6* *Khdrbs2* *Lmx1a* *Lrp2* *Lrp8* *Mea*	*Met* *Mib1* *Mid1* *Msx2* *Neurod4* *Numb* *Pax5* *Pds5a* *Pex13* *Plxnb1* *Plxnb2* *Pole4* *Ptk2* *Ric8a* *Rora* *Shh* *Skor2* *Smad2* *Sun1* *Tmem161b*	*Txnrd1* *Ugt1* *Unc5c* *Vldlr* *Wwox* *Zfp38* *Zfp423* *Zic1* *Zic3*	*Actb* *Ass1* *Atoh1* *Auts2* *Beta2* *Bmpr1a* *Bmpr1b* *Cask* *Ccnd1* *Cdh7* *Cdk5* *Chd7* *Crebbp* *En1* *En2* *Ezh2* *Fgf8* *Foxp1* *Foxp2* *Gbx2*	*Hap1* *Huwe1* *Immp2l* *Itgb1* *Lfg* *Lgl1* *Lmx1a* *Mettl3* *Mthfr* *Mycn* *Nmyc* *Oligo3* *Pdk1* *Rack1* *Reln* *Rere* *Ret* *Skor2* *Slc9a6* *Smad1*	*Smad5* *Sufu* *Talpid3* *Tmem67* *Trsp* *Txnrd2* *Ugt1* *Unc5c* *Wnt5a* *Xrcc1* *Zic1* *Zic4*	*Ahi1* *Barhl1* *Bdnf* *Cadps2* *Dio3* *Dlic* *Gabrb3* *Hdac4* *Itga6* *Mast1* *Nr2c2* *Ntrk2* *Pex14* *Rbm4* *Sacs* *Sam68* *Thrb* *Vav3* *Wwox*	*Ampd2* *Ampd3* *Bcl7a* *Cadm1* *Crmp1* *Delphilin* *Duoxa* *Fzd4* *Gnb5* *Gpc1* *Grp56* *Ip6k3* *Jdp2* *Kipk2* *Klhl1* *Lama1* *Mid1* *Ndph* *Npc* *Nrcam*	*Pcp2* *Prickle2* *Prp* *Pten* *Rgs8* *Rhoa* *Rimbp1* *Tsc2* *Zfp38*
69				52			19	29	

MGI (Mouse Genome Information) Genes are listed in the following categories: abnormal cerebellar cortex morphology (Abnormal fissure, Abnormal lobule, Abnormal vermis morphology: VI and VII) and Small cerebellum (Cerebellum hypoplasia).

PubMed: Genes were retrieved through a keyword search of mouse, knockout, cerebellum, and hypoplasia, and manually classified.

Fifteen genes common between MGI and PubMed (Whole CB reduction and Icf reduction): *Ahi1, Cadps2, Ccnd1, Chd7, En1, En2, Foxp2, Gabrb3, Itgb1, Lmx1a, Skor2, Ugt1, Unc5c, Wwox, Zic1*.

icf, intercrural fissure.

## 7 Signaling pathways critical for cerebellar foliation

Having identified genes essential for cerebellar foliation, we turn to the molecular signaling pathways that affect cerebellar cytoarchitecture. From the 125 genes identified in our MGI and PubMed analyses pertaining to cerebellar hypoplasia and intercrural fissure defects, 28 emerge as key players in developmental signaling, neurotransmission, and cell survival (Table 2). Their disruption profoundly alters cerebellar morphology, often with striking specificity. Here, we explore how core pathways like BMP, Wnt, and Shh, alongside subtle modulators like BDNF, orchestrate foliation and reveal vulnerabilities in developmental disorders (Table 2).

**Table 2:** Genetic disruption of signaling or neurotransmitter pathways causes cerebellar hypoplasia.

Signaling pathways	Whole CB reduction	Icf reduction	References
BMP	*Bmpr1a, Bmpr1b, Smad1, Smad2, Smad5, Zfp423*		Qin et al. (2006), Wang et al. (2011), Tong and Kwan (2013), Warming et al. (2006)
Reelin	*Dab1, Lrp8/Apoer2, Reln, Vldlr*		Trommsdorff et al. (1999), Hong et al. (2000), Yang et al., 2002
Shh	*Sufu*		De Mori et al. (2017)
Wnt	*En1, En2, Rora, Tmem67, Wnt5a*	*Ahi1, Wwox*	Dussault et al. (1998), Sheng et al. (2008), Cheng et al. (2010), Sillitoe et al. (2010), Subashini et al. (2017), Abdelhamed et al. (2019), Cheng et al. (2020)
GABA		*Gabrb3*	DeLorey et al. (2008)
Neurotrophin	*Beta2/Neurod1*	*Barhl1, Bdnf, Cadps2, Ntrk2, Pex14, Vav3*	Gao et al. (1995), Minichiello et al. (1998), Li et al. (2004), Sadakata et al. (2007), Quevedo et al. (2010), Shinoda et al. (2011), Allen et al. (2013), Franks et al. (2023)
Thyroid		*Thrb, Dio3*	Portella et al. (2010), Peeters et al. (2013)

The genes were collected from Pubmed search.

### 7.1 Core developmental signaling pathways

#### 7.1.1 Bone morphogenetic protein signaling

The BMPs are secreted signaling molecules expressed before the formation of cerebellum primordia. BMP signaling is important for the specification of neural stem cells in the anterior rhombic lip, as well as for the generation and differentiation of GCs (Tong et al., 2015). BMPs transduce signals by binding to the BMP receptor (BMRP1/BMPR2) complex. BMPR1 phosphorylates associated R-Smads, which subsequently form a heteromeric complex with co-Smad and translocate into the nucleus for transcriptional regulation. *Bmpr1a/Bmpr1b* double knockout results in severe cerebellar patterning defects (Qin et al., 2006). *ZNF423* mutations are associated with Joubert syndrome (Chaki et al., 2012). Knockout of *Zfp423* impairs cerebellar development (Warming et al., 2006; Alcaraz et al., 2006). Zfp423 is a zinc finger transcription factor that integrates BMP and Notch signaling to regulate the expression of neuronal differentiation factor Hes5 (Masserdotti et al., 2010).

#### 7.1.2 Reelin signaling

Reeler mice with *Reln* mutations exhibit an ataxic gait, partly due to cerebellar underdevelopment. *Dab1* mutations in scrambler mice result in phenotypes similar to those in reeler mice (Cendelin, 2014). Reelin, secreted by GCs in the EGL, controls the migration of posterior-born PCs to form the primordial PCL. As Reelin binds to its receptors apolipoprotein E receptor 2 (ApoER2) and very-low-density lipoprotein receptor (VLDLR), it triggers signal transduction through the adaptor Disabled-1 (Dab1), thereby modulating cytoskeletal rearrangement that direct neuronal migration (Jossin, 2020). Notably, aberrant activation of mTOR signaling results in ubiquitination and destruction of phosphorylated Dab1, and pharmacological inhibition of mTOR restores Reelin-Dab1 signaling and cell migration (Moon et al., 2015).

#### 7.1.3 Wnt signaling

During cerebellar development, Wnt/*β*-catenin activity is present transiently at the embryonic rhombic lip before shifting to the cerebellar ventricular zone, where it promotes neural stem cell proliferation (Selvadurai and Mason, 2011; Pei et al., 2012). Wnt5a knockout leads to cerebellar hypoplasia and the depletion of both GABAergic and glutamatergic neurons (Subashini et al., 2017). Wnt ligands bind to the Frizzled receptors (Fzds) and co-receptors, preventing β-catenin phosphorylation and degradation. Increased β-catenin signaling activates genes including the engrailed transcription factors (EN1/2) that are critical for cerebellar development. Wnt activity is reduced in *Ahi1*-mutant mice, a model of Joubert syndrome (Lancaster et al., 2011). Additionally, Wnt/β-catenin activity is differentially affected by Frizzled-like receptor Tmem67 (Abdelhamed et al., 2019), the oxidoreductase Wwox (Cheng et al., 2020), and retinoid-related orphan receptor RORα (Lee et al., 2010). Notably, these Wnt regulators are implicated in ASD or Joubert syndrome, reinforcing their roles in cerebellar development.

#### 7.1.4 Sonic hedgehog signaling

Shh is secreted by PCs in the ventricular zone and represents the main mitogenic factor driving postnatal GCP expansion in the EGL. In addition, Shh signaling affects Bergmann glial differentiation (De Luca et al., 2016). In the absence of hedgehog ligands, the transmembrane receptor Patched binds and inhibits the activity of Smoothened (Smo). Binding of Shh to Patched activates its downstream signaling. Smo releases the GLI family of transcription factors from sequestration by Suppressor of fused (Sufu), enabling their nuclear translocation and transactivation (De Luca et al., 2016). Gli proteins subsequently promote the expression of genes involved in cell proliferation, such as N-Myc and cyclins (Consalez et al., 2021). Homozygous missense variants in *SUFU* have been identified in Joubert syndrome (De Mori et al., 2017). *Sufu* deficiency causes severe mispatterning of the cerebellum (Kim et al., 2011).

#### 7.1.5 Notch signaling

Notch signaling is also crucial for cerebellar development (Engler et al., 2018). Notch activity maintains the multipotency of cerebellar Sox2^+^ progenitors and its level regulates the ratio of inhibitory to excitatory neuron cell fates from common progenitor cells (Zhang et al., 2021). Notch signaling can be antagonized by the endocytic adaptor Numb. Intriguingly, Numb has multiple splice isoforms that may exert different effects in Notch signaling (Dho et al., 2025). Numb is, however, involved in diverse cellular processes. For example, conditional knockout of *Numb* in PCs impairs motor coordination due to downregulating metabotropic glutamate 1 receptor (mGlu1) on the cell surface (Zhou et al., 2015).

### 7.2 Neurotransmitters, growth factors, and hormones

In addition to the aforementioned factors, cerebellar foliation also involves neurotransmitters and hormones. *γ*-aminobutyric acid (GABA) is primarily known for its role as a synaptic neurotransmitter, but it also regulates cell proliferation, migration, and differentiation during brain development (Owens and Kriegstein, 2002). GABA depolarizes GCPs via ionotropic GABA_A_ receptors and causes their cell cycle exit (Dave and Bordey, 2009). *Gabrb3*-KO mice exhibited deficits in social and exploratory behaviors, concomitant with a reduced intercrural fissure (DeLorey et al., 2008).

#### 7.2.1 Neurotrophins

Neurotrophins represent a group of peptide growth factors including nerve growth factor (NGF), brain-derived neurotrophic factor (BDNF) and neurotrophin-3/4/5 (NT-3/4/5). These signaling molecules bind to their respective Trk family receptors to activate downstream MAP kinase pathways (Allen et al., 2013). In general, neurotrophins function in neuronal differentiation, neuroprotection, and synaptic plasticity. *Bdnf-*deficient mice displayed ataxia, decreased PC complexity and the loss of the intercrural fissure in their cerebellum (Schwartz et al., 1997). BDNF is not crucial for GCP proliferation, but it may contribute to GC maturation and maintenance (Gao et al., 1995). Notably, knockout of *Ntrk2*, which encodes the BDNF/NT-4 receptor TrkB, also caused similar phenotypic changes in the cerebellum as *Bdnf* knockouts (Minichiello et al., 1998). Knockout studies revealed that intercrural fissure formation is also affected by several genes involved in BDNF biogenesis or signaling, including *Cadps2*, *Vav3* and *Pex13/14* (Sadakata et al., 2007; Quevedo et al., 2010; Müller et al., 2011; Abe et al., 2018). Ca^2+^-dependent activator protein 2 (Cadps2) promotes BDNF secretion (Shinoda et al., 2011). Knockout of *Vav3*, a Rac/RhoA guanine nucleotide exchange factor, slightly compromised BDNF/TrkB signaling, but how it regulates BDNF remains unclear (Quevedo et al., 2010). Peroxisome biogenesis deficiency attenuates BDNF–TrkB pathway-mediated development (Müller et al., 2011; Abe et al., 2018). Despite paradoxically elevated levels of BDNF, *Pex14* knockout compromised BDNF–TrkB signaling, likely due to an increase in truncated TrkB (Abe et al., 2018). Besides BDNF, NT-3 and its receptor TrkC are, respectively, regulated by the transcription factors Barhl1 and NeuroD1 (Li et al., 2004; Cho and Tsai, 2006). Interestingly, *Barhl1* knockout also causes intercrural fissure deficiency (Li et al., 2004).

#### 7.2.2 Thyroid hormones

Cerebellar development is sensitive to thyroid hormone levels (Neveu and Arenas, 1996). Hypothyroidism causes cerebellar dysfunction. Biologically active triiodothyronine (T3) binds to intracellular thyroid hormone receptors (TRs) to regulate target genes. Mice harboring ligand-binding mutant *Thrb*∆337 T exhibited reduced intercrural fissure (Portella et al., 2010). Moreover, the level of thyroid hormones can be differently regulated by three iodothyronine deiodinases (Dios). *Dio3* knockout increased the T3 level and, intriguingly, abolished intercrural fissure formation (Peeters et al., 2013). Notably, thyroid deficiency reduces BDNF expression (Chakraborty et al., 2012), suggesting a link between thyroid and BDNF.

**Figure 3:**
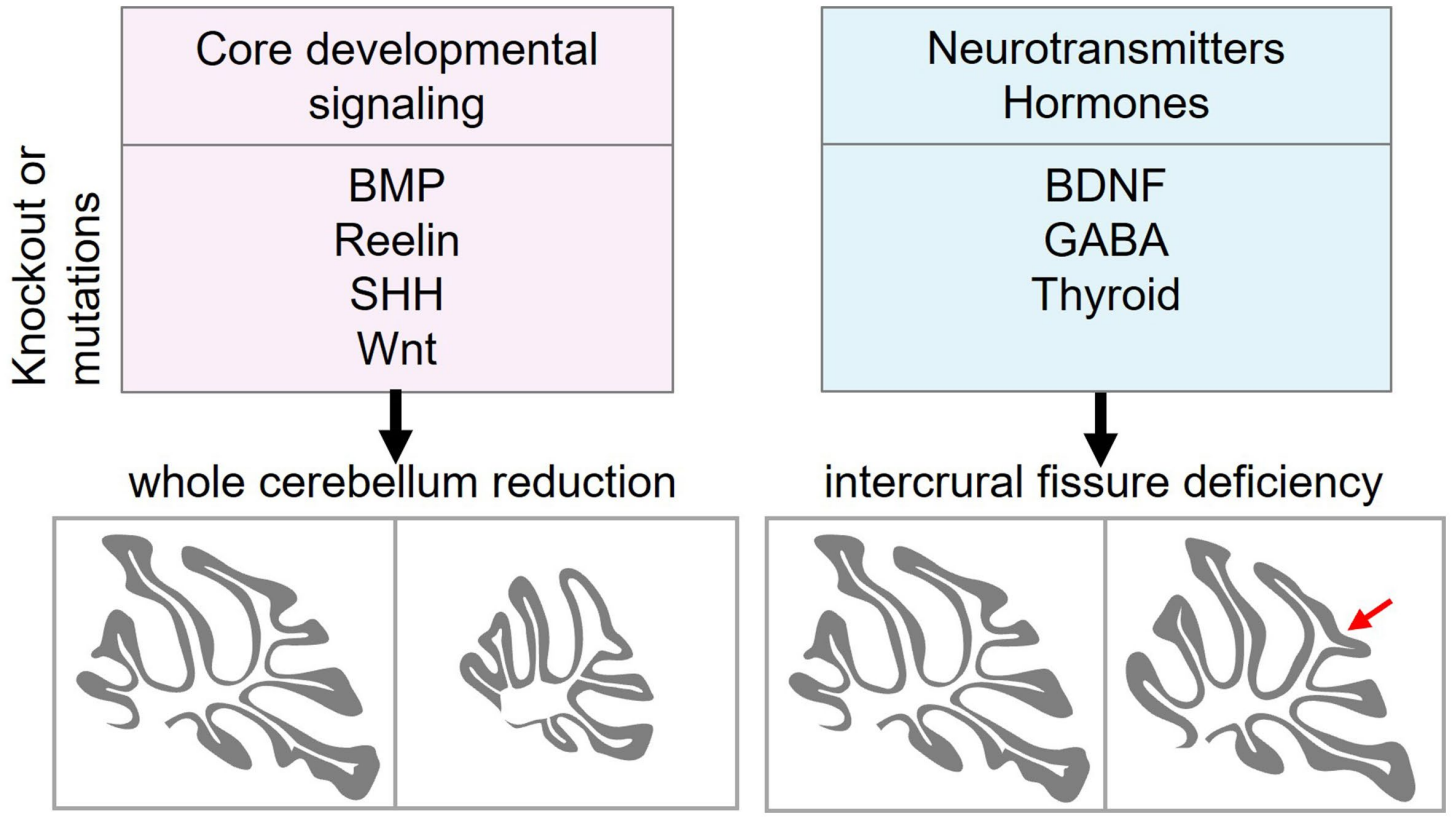
Distinct roles of core developmental signaling and neurotransmitter/hormone signaling in cerebellar morphogenesis. Core developmental signaling pathways, including BMP, Reelin, Shh, and Wnt, regulate the overall growth and patterning of the cerebellum. A Disruption of these pathways reduces the size of the cerebellum. In contrast, ablation of neurotransmitters and hormones such as BDNF (and other neurotrophins), GABA, and thyroid hormone impairs the intercrural fissure (red arrow) formation without affecting gross cerebellar morphology. BMP, bone morphogenetic protein; Shh, sonic hedgehog; BDNF, brain-derived neurotrophic factor; GABA, *γ*-aminobutyric acid.

Our analysis indicated that disruption of core developmental signaling pathways, including BMP, Reelin, Wnt and Shh, substantially impairs cerebellar development, resulting in smaller cerebella. In contrast, ablation of non-core signals, such as BDNF, GABA, and thyroid, disrupts intercrural fissure formation without impairing the overall cerebellar morphogenesis (Figure 3).

## 8 BDNF is crucial for the formation of the fissure between lobules VI and VII

**Figure 4:**
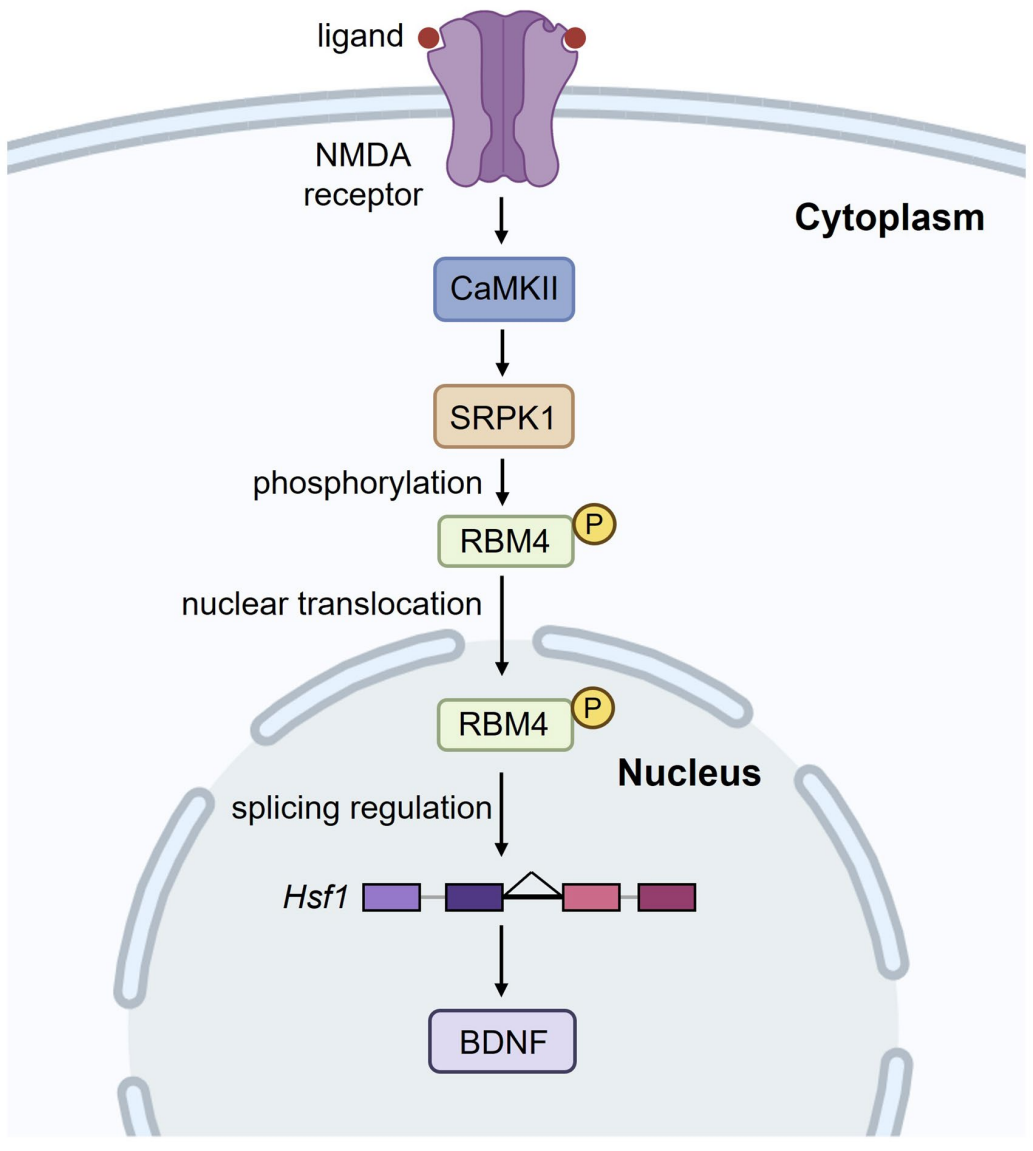
*N*-methyl-d-aspartate (NMDA) receptor activation regulates RBM4-mediated splicing of *Hsf1*. Upon NMDA receptor activation, a signaling cascade involving CaMKII and SRPK1 leads to the phosphorylation of RBM4. Phosphorylated RBM4 translocates into the nucleus, where it regulates the intron excision of *Hsf1*. This, in turn, enhances BDNF expression, which is crucial for neuronal function and development.

Since ASD is associated with abnormalities in vermal lobules VI and VII, identifying the key pathways that shape the intercrural fissure is essential. Although BDNF has been implicated in the formation of the intercrural fissure, strong evidence has been lacking. Strikingly, conventional knockout of two paralogous copies of *Rbm4* gene results in the loss of the intercrural fissure and a significant reduction in BDNF levels (Tsai et al., 2023). RBM4 is an alternative splicing regulator (Markus and Morris, 2009). RNA-seq analysis of *Rbm4* knockout brains revealed intron retention in *Hsf1*, which encodes a transcriptional activator for *Bdnf* (Shen et al., 2024). Intron retention leads to downregulation of HSF1 protein, and hence BDNF reduction. Prenatal re-expression of HSF1 in *Rbm4* knockout brains restored BDNF levels and the intercrural fissure. Similar results were obtained with prenatal supplementation of 7,8-dihydroxyflavone, a TrkB agonist (Tsai et al., 2023), indicating that BDNF plays a crucial role in the formation of the intercrural fissure. Moreover, activation of N-methyl-D-aspartate (NMDA) receptors induces a kinase cascade involving Ca^2+^/Calmodulin-dependent protein kinase II (CaMKII) and SR protein kinase 1 (SRPK1), leading to phosphorylation of RBM4. Phosphorylated RBM4 translocates into the nucleus, where it promotes *Hsf1* intron excision (Figure 4). Such stimulus-activated splicing mechanism is in line with the report that neuronal activation promotes the removal of retained introns (Mazille et al., 2022). It is noteworthy that, unlike *Hsf1* intron excision upon NMDA stimulation, acute stress induces nuclear translocation of HSF1 for *Bdnf* transactivation in the hippocampus (Franks et al., 2023). Thus, developmental cues and cellular stress activate HSF1 via different mechanisms. Taken altogether, BDNF plays a crucial role in intercrural fissure formation. Further studies are needed to determine why this fissure is especially sensitive to BDNF signaling and whether BDNF is crucial for functional circuit formation particularly in the central cerebellar vermis during development.

## 9 Interventions for cerebellar dysfunction

Mouse model with cerebellar hypoplasia can recapitulate key features of human cerebellar disorders (Section 5), making them a viable tool for developing interventions against neurodevelopmental disorders. For example, Joubert syndrome mouse model with defective Wnt signaling exhibit cerebellar midline fusion that can be partially reversed with lithium treatment, an agonist of Wnt signaling (Lancaster et al., 2011). Moreover, a mutation of the glia cell-line derived neurotrophic factor Ret gene causes cerebellar hypoplasia in mice that mimics Down’s syndrome. Such a mutation impairs Shh-mediated development of GCs and glial fibers, and a Smo agonists can rescue these neuronal defects (Ohgami et al., 2021). Mice with mutant methyl-CpG-binding protein 2 gene provide a Rett syndrome model and exhibit deficient BDNF–TrkB activity. A small molecule TrkB agonist, LM22A-4, can alleviate the motor learning deficits in these mice (Medeiros et al., 2024). Cerebellar BDNF expression is reduced in postmortem SCA6 human tissues (Takahashi et al., 2012). Consistent with this finding, reduced TrkB–BDNF signaling is evident in the early disease stage of a SCA6 mouse model (SCA6^84Q/84Q^). Prolonged administration of 7,8-dihydroxyflavone improved ataxic phenotypes and PC firing rate (Cook et al., 2022). As described above, prenatal administration of 7,8-dihydroxyflavone restored cerebellar development and motor learning in *Rbm4* knockout mice (Tsai et al., 2023). Given the effects of HSF1 overexpression in *Rbm4* knockout mice (Shen et al., 2024), using small-molecule compounds to activate HSF1 for intervention is possible. HSF1 is targeted by multiple stress-induced signaling cascades (Hooper et al., 2016). Tanespimycin (17-AAG), a derivative of the antibiotic geldanamycin, can de-repress HSF1 from sequestration by its molecular chaperone HSP90 (Chen et al., 2014). 17-AAG can restore synaptic protein levels such as PSD95 and BDNF in Alzheimer’s disease models (Chen et al., 2014). Therefore, it may be possible to treat developmentally disordered brains with low BDNF, such as those with *Rbm4* knockout, with 17-AAG or similar functional molecules. These findings suggest that pharmacological restoration of signaling activities may be useful for treating developmental disorders in the future.

## 10 Conclusion

This review summarizes key developmental signaling pathways and neuromodulators involved in cerebellar development. Dysregulation of these pathways results in cerebellar malformation, ranging from hypoplasia to local foliation defects. It is noteworthy that BDNF plays a pivotal role in shaping the intercrural fissure between lobules VI-VII—a structure implicated in ASD. Prenatal restoration of BDNF biogenesis or signaling can prevent cerebellar deficits in BDNF deficient mouse models. Additionally, it is possible to reverse other BDNF-deficient-caused deficits in the cerebellum through activation of TrkB. Unraveling how these pathways converge across species and disorders promises deeper insights into cerebellar morphogenesis and innovative therapies for neurodevelopmental challenges.

## Footnotes

^1^ www.informatics.jax.org

^2^ pubmed.ncbi.nlm.nih.gov
